# The Dentin Sialoprotein (DSP) Domain Regulates Dental Mesenchymal Cell Differentiation through a Novel Surface Receptor

**DOI:** 10.1038/srep29666

**Published:** 2016-07-19

**Authors:** Chunyan Wan, Guohua Yuan, Daoshu Luo, Lu Zhang, Heng Lin, Huan Liu, Lei Chen, Guobin Yang, Shuo Chen, Zhi Chen

**Affiliations:** 1 State Key Laboratory Breeding Base of Basic Science of Stomatology (Hubei-MOST) and Key Laboratory for Oral Biomedicine of Ministry of Education (KLOBM), School and Hospital of Stomatology, Wuhan University, Wuhan, 430079, China; 2 Department of Developmental Dentistry, University of Texas Health Science Center, San Antonio, Texas, 78229-3700, United States; 3 Department of Anatomy, Histology and Embryology, School of Basic Medical sciences, Fujian Medical University, Fuzhou, 350108, China; 4 Department of Surgery, The First Affiliated Hospital, Fujian Medial University, Fuzhou, 350005, China

## Abstract

Dentin sialophosphoprotein (DSPP) is a dentin extracellular matrix protein that is processed into dentin sialoprotein (DSP), dentin glycoprotein (DGP) and dentin phosphoprotein (DPP). DSP is mainly expressed in odontoblasts. We hypothesized that DSP interacts with cell surface receptors and subsequently activates intracellular signaling. Using DSP as bait for screening a protein library, we demonstrate that DSP acts as a ligand and binds to integrin β6. The 36 amino acid residues of DSP are sufficient to bind to integrin β6. This peptide promoted cell attachment, migration, differentiation and mineralization of dental mesenchymal cells. In addition, DSP ^aa183-219^ stimulated phosphorylation of ERK1/2 and P38 kinases. This activation was inhibited by an anti-integrin β6 antibody and siRNA. Furthermore, we demonstrate that this DSP fragment induces SMAD1/5/8 phosphorylation and nuclear translocation via ERK1/2 and P38 signaling. SMAD1/5/8 binds to SMAD binding elements (SBEs) in the DSPP gene promoter. SBE mutations result in a decrease in DSPP transcriptional activity. Endogenous DSPP expression was up-regulated by DSP ^aa183-219^ in dental mesenchymal cells. The data in the current study demonstrate for the first time that this DSP domain acts as a ligand in a RGD-independent manner and is involved in intracellular signaling via interacting with integrin β6. The DSP domain regulates DSPP expression and odontoblast homeostasis via a positive feedback loop.

During the process of dentinogenesis, highly controlled extracellular events occur. This process is tightly controlled by odontoblasts, which secrete extracellular matrix (ECM) proteins and regulate dentin mineralization. ECM comprises collagenous and non-collagenous proteins (NCPs)^1^,^2^. Among NCPs, dentin sialophosphoprotein (DSPP) is the most abundant ECM in dentin and is processed into three major forms: dentin sialoprotein (DSP), dentin glycoprotein (DGP) and dentin phosphoprotein (DPP)^3^. Among them, DSP and DPP are chiefly expressed in odontoblasts and dentin^4^,^5^. Both DSP and DPP play unique roles in dentinogenesis^6^. Mutations of either the DSP or DPP domain cause dentinogenesis imperfecta type II and III (DGI-II and III) and dentin dysplasia type II (DD-II), the most common dentin genetic disorder^7^,^8^,^9^,^10^,^11^. DSP is a sialic acid-rich, glycosylated protein^1^ and is involved in the initiation of dentin mineralization^6^,^12^,^13^, whereas DPP contains abundant aspartic acid and serine, comprising approximately 70–80% of the total amino acid residues^2^, and facilitates the maturation of dentin^14^.

DSPP is a member of the SIBLING (Small Integrin-Binding Ligand N-linked Glycoproteins) family, consisting of bone sialoprotein (BSP), dentin matrix protein1 (DMP1), DSPP, osteopontin (OPN), and matrix extracellular phosphoglycoprotein (MEPE). These SIBLING genes are clustered on human chromosome 4^15^,^16^,^17^,^18^,^19^,^20^ and share an Arg-Gly-Asp (RGD) sequence that facilitates cell attachment, migration, differentiation and triggers intracellular signal transduction via binding to cell surface receptors, such as integrin^21^. For example, the RGD motif within DMP1 regulates osteoblast differentiation by interacting with integrin αvβ3 and then activating ERK, JNK and P38 MAPK signaling in human preosteoblasts^22^,^23^,^24^. OPN propagates signals by binding to integrin αvβ1, αvβ3 and αvβ5^25^,^26^. In mouse DSPP, RGD is located within the DPP domain, and DPP activates MAPK and SMAD pathways and triggers intracellular signals by directly interacting with integrin^27^,^28^. By contrast, DSP does not contain any RGD domains^9^. Evidence suggests that DSP and peptides derived from DSP regulate gene expression and protein phosphorylation and induce dental primary/stem cell differentiation^29^,^30^. However, the molecular mechanisms of the DSP control of gene expression and cell differentiation are not well understood.

Integrins are a family of cell surface proteins that mediate cell-to-cell and cell-to-extracellular matrix interactions. They consist of two subunits: α and β^31^. Many, but not all integrins, bind to ligands, such as RGD, forming the RGD-integrin complex. This complex facilitates intracellular signal transduction during physiological and pathological activities^17^,^32^,^33^.

Based on the above description, we hypothesized that DSP acts as a ligand, regulates intracellular signal transductions and promotes dental mesenchymal cell differentiations via its receptor (s). Here, we found that DSP is capable of binding to its cell surface receptor, integrin β6. Further analyses revealed that the 36 amino acids of the DSP domain interact with integrin β6 and stimulate cell attachment, spreading, migration and differentiation of dental mesenchymal cells. DSP-associated mechanisms induce phosphorylation of ERK1/2, P38 and SMAD1/5/8. SMAD1/5/8 combined with SMAD4 binds to SMAD binding elements (SBEs) in the DSPP gene regulatory region and activates DSPP gene transcription and cell behaviors.

## Results

### DSP ^aa 183-219^ binds to integrin β6

**Figure 1:**
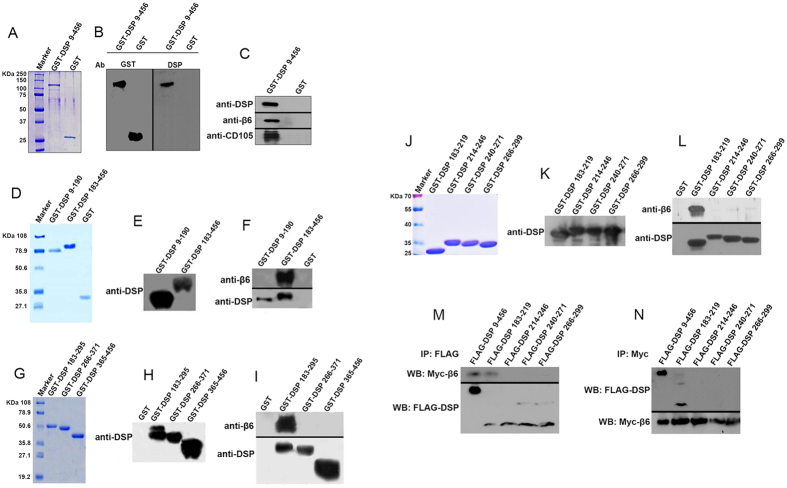
DSP ^aa 183-219^ binds to integrin β6. Recombinant DSP 9–456, 9–190, 183–456, 183–295, 266–371, 365–456, 183–219, 214–246, 240–271, and 266–299 were expressed in *Escherichia coli* BL21 and purified according to the manufacturer’s instruction as described by “Materials and methods”. The DSP number starts at the translational start site of DSPP (Met) as No.1. The purified DSP fusion proteins were confirmed by Coomassie blue staining (**A**,**D**,**G**,**J**) and western blot assays using either an anti-GST or anti-DSP antibody (**B**,**E**,**H**,**K**). The GST-DSP fusion protein was incubated with cell lysis from mouse odontoblast-like cells in the lysis buffer overnight at 4 °C. After the reaction, the glutathione agarose beads were added for further incubation. The samples were centrifuged, and the supernatant was removed. After extensive washes, the DSP binding proteins were eluted by reduced glutathione. The eluted samples were mixed with 2 × SDS-PAGE gel loading buffer, run on SDS-PAGE gels and analyzed by western blot assay. Interaction between DSP polypeptides and integrin β6 by GST pull down was detected by western blot using anti-DSP and anti-β6 antibodies (**C**,**F**,**I**,**L**). For *in vivo* studies, DSP cDNA was subcloned into a CMV mammalian expression plasmid tagged with FLAG peptides, whereas β6 cDNA was subcloned into a CMV-Myc vector. Both plasmids were transiently transfected into 293T cells for 48 h. The proteins were harvested, and protein-protein interactions were immunoprecipitated using either a FLAG or Myc antibody followed by western blotting with an anti-FLAG or anti-Myc antibody (**M**,**N**).

To assess whether DSP is capable of interacting with other proteins, we generated a GST-DSP fusion protein (Fig. 1A,B). The DSP fusion protein was used as bait to screen a protein library isolated from mouse odontoblast-like cells. Co-IP assays revealed that four proteins among 110 candidates interacted with DSP, including integrin β6 (Fig. 1C). To further identify the specific DSP domain interacting with integrin β6, the NH_2_-terminal-DSP ^aa9-190^ and COOH-terminal-DSP ^aa183-456^ domains were expressed, purified and confirmed by Coomassie blue staining and western blot assays (Fig. 1D,E). Protein-protein interaction assays revealed that integrin β6 could bind to the COOH-terminal fragment of DSP ^aa183-456^, but not the NH_2_-terminal domain ^aa9-190^ (Fig. 1F). To narrow the binding size of DSP, three small fragments of the COOH-terminal DSP domain were generated and confirmed by Coomassie blue staining and western blot assays (Fig. 1G,H). IP assays revealed that integrin β6 was bound by the 112 amino acid residues ^aa183-295^ of DSP, but not the other two fragments (Fig. 1I). Then, COOH-terminal DSP ^aa183-295^ was further divided into four fragments: DSP ^aa183-219^, DSP ^aa214-246^, DSP ^aa240-271^ and DSP ^aa266-299^ (Fig. 1J,K). Protein-protein interaction assays indicated that only 36 amino acid residues of the DSP domain ^aa183-219^ could bind to integrin β6 (Fig. 1L). To further determine whether DSP ^aa183-219^ binds to integrin β6 *in vivo*, different DSP fragments were subcloned into a mammalian expression vector tagged with FLAG, whereas the integrin β6 gene was subcloned into a mammalian expression vector tagged with Myc. Both of the expression vectors were transfected into 293T cells. Co-IP assays revealed that integrin β6 was able to bind to DSP ^aa183-219^, but not other DSP regions (Fig. 1M,N). This result demonstrates that DSP acts as a ligand and interacts with its cell membrane receptor, integrin β6.

### DSP ^aa183-219^ promotes attachment, spreading and migration of mouse dental papilla mesenchymal cells

**Figure 2:**
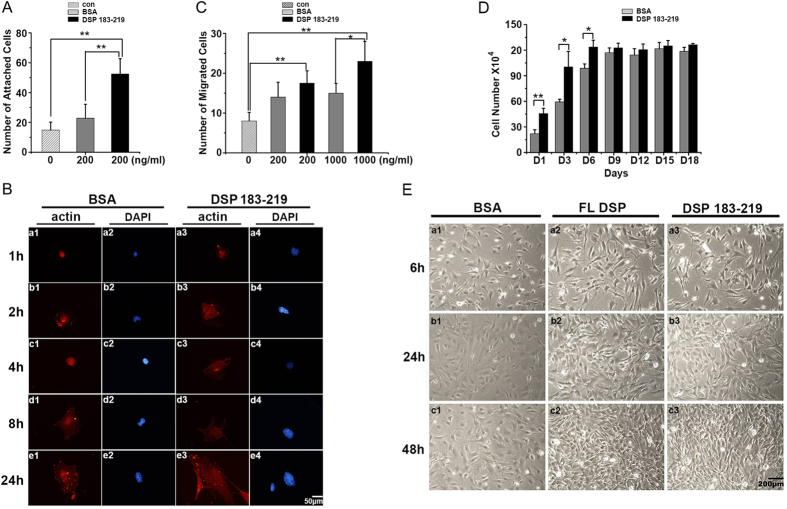
DSP ^aa183-219^ promotes attachment, spreading, proliferation and migration of mouse dental papilla mesenchymal cells. (**A**) mDPC6T cells were seeded on DSP ^aa183-219^- or BSA-coated plates. The adherent cells were fixed, stained and counted; (**B**) mDPC6T cells were cultured with or without 200 ng/ml DSP ^aa183-219^ for 1, 2, 4, 8, or 24 h. The cells were then fixed and stained for actin; (**C**) cells were cultured with or without 200 ng/ml or 1 μg/ml DSP ^aa183-219^ as a chemoattractant. After 12 h of incubation, the filters were fixed and stained with 0.1% (w/v) crystal violet; (**D**) cells were treated with or without 200 ng/ml of DSP ^aa183-219^. The cell number was directly counted at the time point of day 1, 3, 6, 9, 12, 15 and 18; (**E**) cell morphology was observed under microscope after 6, 24, and 48 h of incubation with or without DSP ^aa183-219^.

To study the biological roles of DSP ^aa183-219^ in mouse immortalized dental papilla mesenchymal cells (mDPC6T)^34^, we first evaluated the effect of this DSP peptide on cell attachment. We observed that after 12 h of incubation, the number of attached cells grown on 200 ng/ml of DSP ^aa183-219^-treated plates was approximately two-fold increased compared with BSA-treated plates (*P* < 0.01) and approximately three-fold increased compared with non-treated plates (*P* < 0.01) (Fig. 2A). The results indicate that DSP ^aa183-219^ is sufficient to promote mDPC6T cell attachment.

We then investigated whether DSP ^aa183-219^ has an effect on mDPC6T cell spreading. mDPC6T cells were seeded on cover-glass slides coated with or without 200 ng/ml DSP ^aa183-219^ and cultured for various time points. The cells were then fixed and stained for actin. We found that the spreading area of cells seeded on DSP ^aa183-219^-coated cover glass slides was larger, especially at 24 h of induction. However, cells cultured on BSA-coated cover glass slides appeared round, and the cells exhibited small spreading areas at all time points (Fig. 2B). The results show that DSP ^aa183-219^ promotes spreading of mouse dental papilla mesenchymal cells.

We further examined the effect of DSP ^aa183-219^ on cell migration. mDPC6T cells were stimulated with different concentrations of DSP ^aa183-219^ or BSA as a control for 12 h. We found that both 200 ng/ml and 1 μg/ml DSP ^aa183-219^ increased cell migration. In the presence of 200 ng/ml DSP ^aa183-219^, the number of migrated cells was greater than either the unstimulated cells (*P* < 0.05) or the BSA-treated cells. In the presence of 1 μg/ml DSP ^aa183-219^, the number of migrated cells was approximately 4-fold increased compared with non-stimulated cells and approximately doubled compared with BSA-treated cells (Fig. 2C).

### DSP ^aa183-219^ stimulates mDPC6T cell proliferation and differentiation

**Figure 3:**
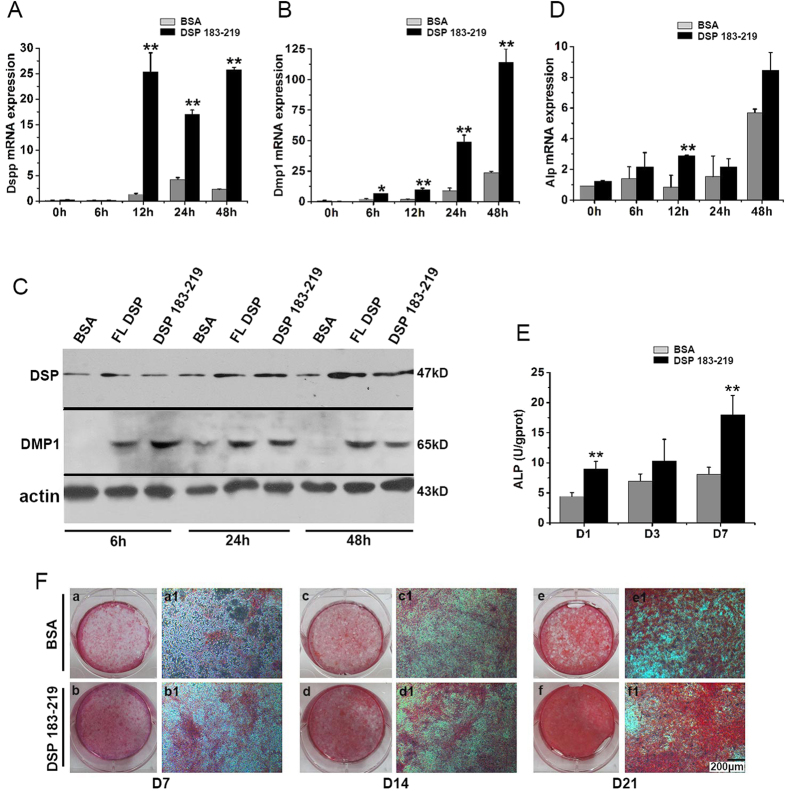
DSP ^aa183-219^ stimulates mDPC6T cell differentiation and mineralization. DSPP (**A**), DMP1 (**B**), ALP (**D**) mRNA expression of mDPC6T cells treated with or without 200 ng/ml DSP ^aa183-219^ for 0, 6, 12, 24 and 48 h. Data are expressed as the mean ± S.D. for 3 independent experiments. Means are considered significantly different by an independent *t-*test if *P* < 0.05. *P < 0.05; **P < 0.01; (**C**) western blot analysis of DSP and DMP1 in mDPC6T cells treated with or without 200 ng/ml DSP ^aa183-219^ for 6, 26 and 48 h with anti-DSP or anti-DMP1 antibodies; (**E**) ALP activity of mDPC6T cells treated with or without 200 ng/ml of DSP ^aa183-219^ in calcifying medium for 1, 3, and 7 days; (**F**) alizarin red staining of mDPC6T cells treated with or without 200 ng/ml of DSP ^aa183-219^ in calcifying medium for 7, 14, and 21 days.

We examined the functional roles of DSP ^aa183-219^ in mDPC6T cell proliferation and differentiation. When mDPC6T cells were cultured for 1, 3, 6, 9, 12, 15 and 18 days in the presence of DSP ^aa183-219^, the DSP peptide promoted mDPC6T cell growth in a time-dependent manner. The cell growth number peaked on day 9 of culture and then remained approximately the same thereafter in the treated and untreated groups (Fig. 2D). Cell morphology was observed with or without DSP peptide treatment using a light microscope. The cell morphology remained unchanged after 6 h of treatment with either full-length DSP or DSP ^aa183-219^ compared with the control group. However, cells cultured with DSP ^aa183-219^ or full-length DSP after 24 and 48 h of induction formed clusters, whereas cells without the DSP treatment retained a fibroblast-like morphology (Fig. 2E). When most dental mesenchymal cells differentiate into putative odontoblasts, they form clusters and increase the expression of odontoblast differentiation markers^35^,^36^. We then examined the DSPP and DMP1 gene expression levels given that these two genes are important markers of odontoblast differentiation^1^. These results showed that DSP ^aa183-219^ stimulated expression of DSPP, as detected by qRT-PCR and western blot analyses (Fig. 3A,C), after 12 h of induction, whereas the fragment upregulated the expression of DMP1 after 6 h of stimulation (Fig. 3B,C). Given that ALP is an important marker for bone and dental cell differentiation^37^, we then tested the ALP levels in the DSP ^aa183-219^-treated group. We observed that this DSP fragment up-regulates expression of ALP in a time-dependent manner (Fig. 3D,E). We also detected mineralization deposits in cells treated with or without DSP ^aa183-219^ in mineralization culture medium for 7, 14, and 21 days. Increased mineralization deposits were noted in the DSP ^aa183-219^-treated group compared with the control groups at the time points examined (Fig. 3F).

### DSP ^aa183-219^ promotes phosphorylation of P38, ERK1/2, and SMAD1/5/8 and nuclear translocation of SMAD1/5/8

**Figure 4:**
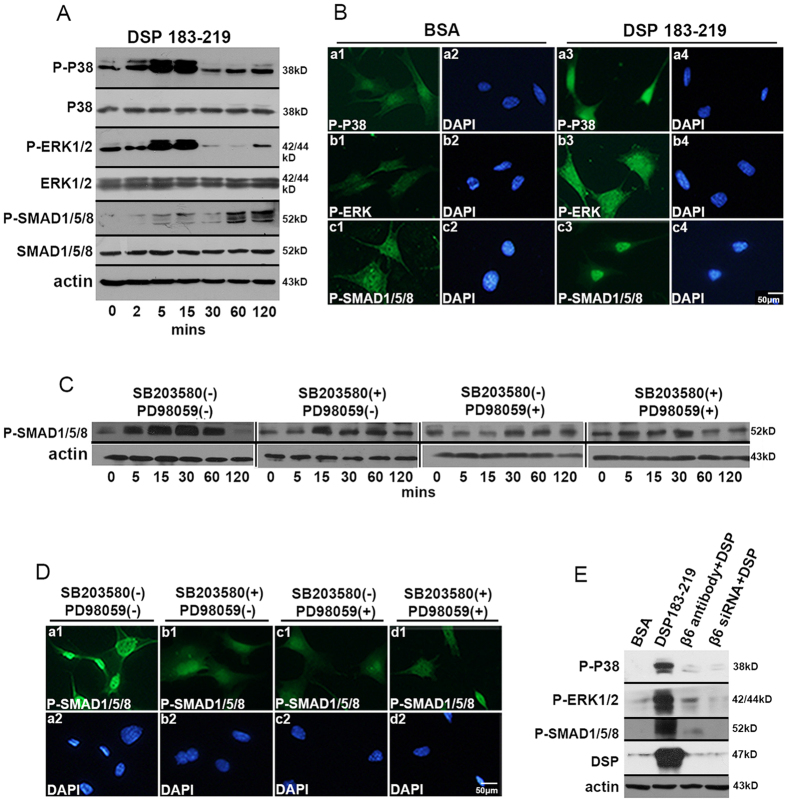
DSP-β6 interaction activates P38, ERK1/2, and SMAD1/5/8 phosphorylation and the nuclear translocation of SMAD1/5/8. (**A**) Western blot analysis of phospho-P38, phospho-ERK1/2, and phospho-SMAD1/5/8 of mDPC6T cells subject to DSP ^aa183-219^ stimulation for 0, 2, 5, 15, 30, 60, and 120 min. (**B**) Immunofluorescence assay of phospho-P38, phospho-ERK1/2, and phospho-PMAD1/5/8 of mDPC6T with or without DSP ^aa183-219^ stimulation. (**C**) Western blot analysis of phospho-SMAD1/5/8 in mDPC6T cells treated with or without 20 μM SB203580 and/or 100 μM PD98059 for 1 h followed by 200 ng/ml DSP ^aa183-219^ for 0, 5, 15, 30, 60, and 120 min. (**D**) Immunofluorescence analysis of phospho-SMAD1/5/8 in mDPC6T cells treated with or without 20 μM P38 SB203580 and/or 100 μM PD98059 followed by 200 ng/ml DSP ^aa183-219^. (**E**) mDPC6T cells were treated with or without DSP ^aa183-219^ for 15 min; treated with 25 μg/ml anti-integrin β6 antibody for 1 h followed by 200 ng/ml DSP ^aa183-219^ for 15 min; or treated with 80 nM integrin siRNA for 24 h followed by 200 ng/ml DSP ^aa183-219^ for 15 min. Phospho-P38, phospho-ERK1/2, phospho-SMAD1/5/8 and DSP were analyzed with anti-phospho-P38, anti-phospho-ERK1/2, anti-phospho-SMAD1/5/8 and anti-DSP antibodies.

To determine the effect of DSP ^aa183-219^ on intracellular signal transduction through integrin β6, mDPC6T cells were treated with DSP ^aa183-219^ at different time periods. Western blot assay showed that this DSP peptide stimulated phosphorylation of P38, ERK1/2 and SMAD1/5/8. The expression levels of phospho-P38 and phospho-ERK1/2 were significantly increased in mDPC6T cells after 2 min of DSP ^aa183-219^ treatment. The maximal phosphorylation of P38 and ERK1/2 occurred at 15 min of induction, whereas SMAD1/5/8 phosphorylation was increased later than P38 and ERK1/2 phosphorylation. SMAD1/5/8 phosphorylation was noted at 5 min and peaked at 1 h after DSP ^aa183-219^ induction (Fig. 4A). Immunofluorescence data indicated that phospho-SMAD1/5/8 significantly accumulated in the nuclei of mDPC6T cells after DSP ^aa183-219^ treatment, whereas phospho-SMAD1/5/8 was distributed in both the nucleus and cytoplasm in the control group. In addition, high and intense phospho-P38 and phospho-ERK1/2 expression was noted in the DSP ^aa183-219^-treated group in mDPC6T cells compared with the control group (Fig. 4B).

### Phosphorylation of P38, ERK1/2 and SMAD1/5/8 is inhibited by integrin β6 inhibitors

We further determined whether the DSP-integrin signal regulates SMAD1/5/8 phosphorylation via the MAP kinase pathway. mDPC6T cells were treated with 20 μM P38 inhibitor (SB203580) and/or 100 μM ERK inhibitor (PD98059) for 1 h followed by the addition of 200 ng/ml DSP ^aa183-219^ for different time periods. The results showed that SMAD1/5/8 phosphorylation was inhibited by P38 or/and ERK inhibitors (Fig. 4C). Immunofluorescence data demonstrated that these P38 (SB203580) and ERK (PD98059) inhibitors could effectively block phosphorylation and nuclear translocation of SMAD1/5/8 induced by DSP ^aa183-219^ (Fig. 4D). Next, we verified whether DSP affected P38, ERK1/2 and SMAD1/5/8 phosphorylation via integrin β6 signaling. mDPC6T cells were treated with DSP ^aa183-219^ plus or minus an anti-integrin β6 antibody or integrin β6 siRNA. Figure 4E shows that DSP ^aa183-219^ induction of P38, ERK1/2 and SMAD1/5/8 phosphorylation was dramatically inhibited by either an anti-integrin β6 antibody or integrin β6 siRNA. More interestingly, endogenous DSP expression induced by DSP ^aa183-219^ in the mouse dental papilla mesenchymal cells was also inhibited by an anti-integrin β6 antibody and integrin β6 siRNA. These results suggest that DSP ^aa183-219^ promotes P38, ERK1/2 and SMAD1/5/8 phosphorylation and DSP expression via integrin β6 signaling. These data indicate that the DSP-integrin β6 complex stimulates the phosphorylation and nuclear translocation of SMAD1/5/8 mediated by P38 and ERK1/2 kinases.

### Cell proliferation and cell differentiation are induced by P38-ERK1/2-SMAD1/5/8 after DSP-β6 interaction

**Figure 5:**
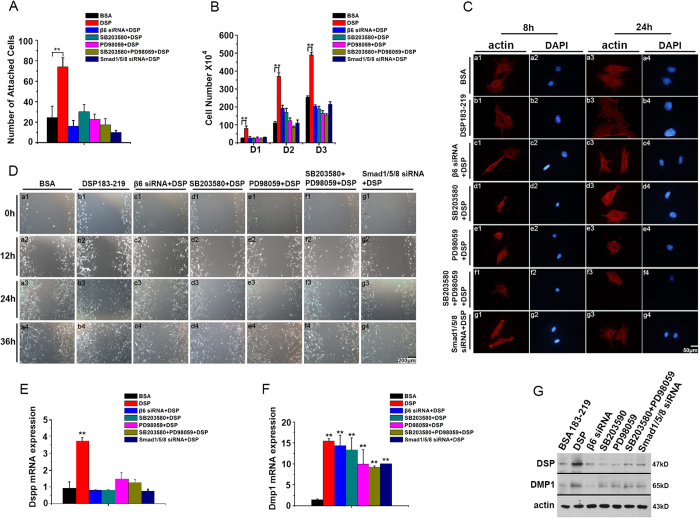
Cell proliferation and differentiation were induced by P38-ERK1/2-SMAD1/5/8 after DSP-β6 interaction. mDPC6T cells were pretreated with or without β6 siRNA (80 nM) for 24 h, SB203580 (20 uM) for 1 h, PD98059(20 uM) for 1 h, or SMAD1/5/8 siRNA (80 nM) for 24 h. (**A**) Cells were then seeded onto DSP-coated plates. The attached cells were fixed, stained and counted. (**B**) cells were then treated with 200 ng/ml of DSP ^aa183-219^. The cell number was directly counted at the different time points on days 1, 2, and 3. (**C**) mDPC6T cells were seeded on 200 ng/ml DSP ^aa183-219^-coated cover glass and cultured for 8 and 24 h. The cells were then fixed and stained for actin. (**D**) cells were cultured with 1 μg/ml DSP ^aa183-219^ as a chemoattractant and then fixed and observed at 0, 12, 24 and 36 h. DSPP (**E**), DMP1 (**F**) mRNA expression in mDPC6T cells treated with 200 ng/ml DSP ^aa183-219^ for 48 h. Data are expressed as the mean ± S.D. for 3 independent experiments. Means are considered significantly different by independent *t* test if *P* < 0.05. *P < 0.05; **P < 0.01; (**G**) western blot analysis of DSP and DMP1 of mDPC6T cells treated with 200 ng/ml DSP ^aa183-219^ for 48 h with anti-DSP or anti-DMP1 antibodies.

To study the correlation between the P38-ERK1/2-SMAD1/5/8 signaling pathway and DSP-induced cell behavior changes, mDPC6T cells were separately pretreated with 80 nM integrin siRNA for 24 h, 20 μM SB203580 and/or 100 μM PD98059 for 1 h, or 80 nM SMAD1/5/8 siRNA for 24 h followed by a 200 ng/ml DSP ^aa183-219^ induction. Cell attachment (Fig. 5A), cell proliferation (Fig. 5B), cell spreading (Fig. 5C), and cell migration (Fig. 5D) induced by this DSP peptide were inhibited by the above compounds. Additionally, we observed that DSP ^aa183-219^-induced DSPP and DMP1 expression was inhibited (Fig. 5E–G). Taken together, these data indicate that DSP ^aa183-219^ interacts with integrin β6 and promotes cell attachment, spreading, proliferation, differentiation and migration as well as induction of DSPP and DMP1 gene expression in dental papilla mesenchymal cells through P38-ERK1/2-SMAD1/5/8 signaling.

### DSP up-regulates DSPP gene transcription through SMAD1/5/8

**Figure 6:**
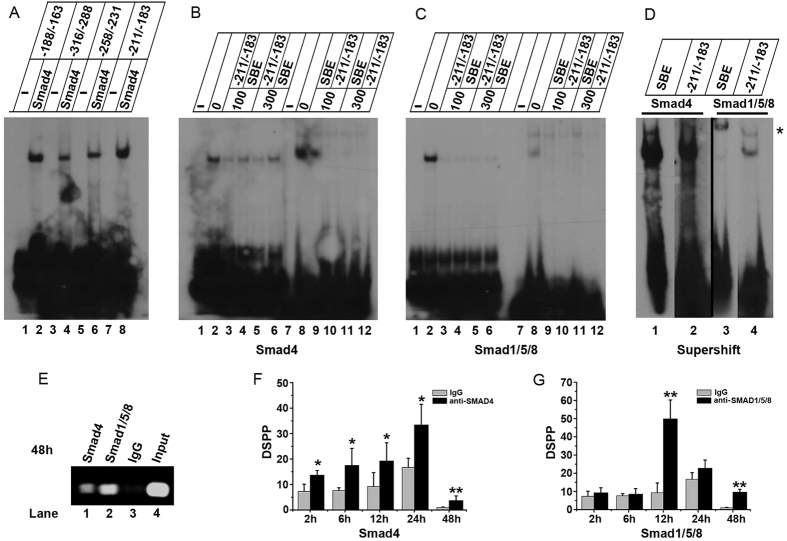
SMAD4 and SMAD1/5/8 bind to SBEs in mDSPP promoter region. (**A**) ^32^P-labeled double stranded DSPP probes were incubated with 5 μg of SMAD4 overexpressing cell nuclear extracts. The sequences selected for DSPP probes are listed below: -316/-288 (GCAGGGTGACAGA GTCTAAGTGGCTCTTT), -258/-231 (TAAGACACAAAACAGTCTTCCAGGAGCT), -211/-183 (AGTCTAGTCCTTTTGGAACCAAAGGTCT), and -188/-163 (GGTCTCAGTGAGCCAACGTAC CGGCG). Competition analysis of the SMAD4-DSPP (**B**) and SMAD1/5/8-DSPP (**C**) complex formation with 100- or 300- fold excesses of standard SBE or unlabeled competitors. (**D**) supershift analysis of SMAD4-DSPP and SMAD1/5/8-DSPP complex with anti-SMAD4 and anti-PSMAD1/5/8 antibodies. (**E**) ChIP analysis of SMAD4-DSPP and SMAD1/5/8-DSPP binding after SMADs and DSPP promoter region were overexpressed in 293T for 48 h; qualitative analysis of SMAD4 (**F**) and SMAD1/5/8 (**G**) binding to the DSPP promoter region at 2, 6, 12, 24, and 48 h with ChIP.

As described above, DSP ^aa183-219^ facilitates dental papilla mesenchymal cell differentiation and DSPP gene expression (Figs 4 and 5). The DSPP gene is an important marker for dental mesenchymal cell differentiation and is involved in odontoblast homeostasis^33^. In addition, DSP ^aa183-219^ induces SMAD1/5/8 phosphorylation and nuclear translocalization. SMAD1/5/8 are transcription factors and control the expression of their down-stream genes^32^. Therefore, we studied whether DSP ^aa183-219^ positively regulates DSPP gene expression and dental mesenchymal cell differentiation via the SMAD signal pathway. The mouse DSPP gene regulatory region was analyzed to search for potential SMAD binding elements (SBEs) using the Transcription Element Search System (TESS) (http://www.cbil.upenn.edu/cgi-bin/tess/tess). Four SBEs were identified in the proximal promoter of the DSPP gene. To determine if SMAD proteins bind to these SBEs in the DSPP promoter region, Electrophoretic mobility shift assay (EMSA) was performed. EMSA revealed that the four SBEs were bound by nuclear extracts from SMAD4 overexpression cells (Fig. 6A). Given that the nucleotide sequence between -211 and -183 of the mouse DSPP promoter region is highly homologous with that of the human promoter (data not shown), we chose this nucleotide sequence for further study. We observed that the SBEs in the DSPP promoter and SBE consensus sequences were bound by SMAD4 overexpression cell nuclear extracts. Competition assays revealed that the SMAD4 protein-DNA complex at the DSPP promoter was competed away by 100- and 300-fold molar excesses of the unlabeled homologous element and SBE consensus oligonucleotides and vice versa (Fig. 6B). In addition, the SMAD1/5/8 protein is able to directly bind to this element. The SMAD1/5/8 protein-DNA complex was completely competed away by 100- and 300- fold molar excesses of either the unlabeled homologous element or SBE consensus oligonucleotide (Fig. 6C).

Using anti-SMAD4 and anti-PSMAD1/5/8 antibodies, we performed a super-shift assay. Incubation of the nuclear extracts with either the anti-SMAD4 or anti-PSMAD1/5/8 antibody, the labeled double-stranded sequence from -211 to -183 and the SBE consensus probes led to the formation of slower migrating protein-DNA complexes, whereas the nonspecific serum had no effect (Fig. 6D). These results verify that both SMAD4 and SMAD1/5/8 bind to -211 to -183 in the DSPP promoter *in vitro*.

To further assess whether SMAD4 or SMAD1/5/8 binds to the DSPP promoter region from -211 to -183 *in vivo*, we performed a ChIP assay. Cells were transfected with pcDNA-SMAD4, pCMV-SMAD1, pCMV-SMAD5, pcDNA-SMAD8, and pGL-Luc-mDSPP-500/+54 expression vectors. After 48 h, anti-SMAD4 or anti-PSMAD1/5/8 antibody-immunoprecipitated protein-DNA complexes were crosslinked, and the purified DNA was used as a template for PCR using primers covering the DSPP gene -211 to -183 region. As expected, 214-bp PCR products were amplified from the DNA fragment immunoprecipitated by anti-SMAD4 and anti-PSMAD1/5/8 antibodies, whereas no clear PCR product was amplified by IgG as a negative control (Fig. 6E). To measure the binding affinity of SMAD4 or SMAD1/5/8 to the SBEs in the DSPP gene promoter at different time periods, a ChIP assay was performed to detect protein-DNA complexes 2, 6, 12, 24 and 48 h after transfection. Maximal binding of SMAD4 to its binding site occurred at 12 h after transfection, whereas SMAD5 binding to the SBE in the DSPP promoter peaked 24 h after transfection (Fig. 6F,G). These results indicate that SMAD4 and SMAD1/5/8 directly bind to the mouse DSPP promoter region from -211 to -183 *in vivo*.

**Figure 7:**
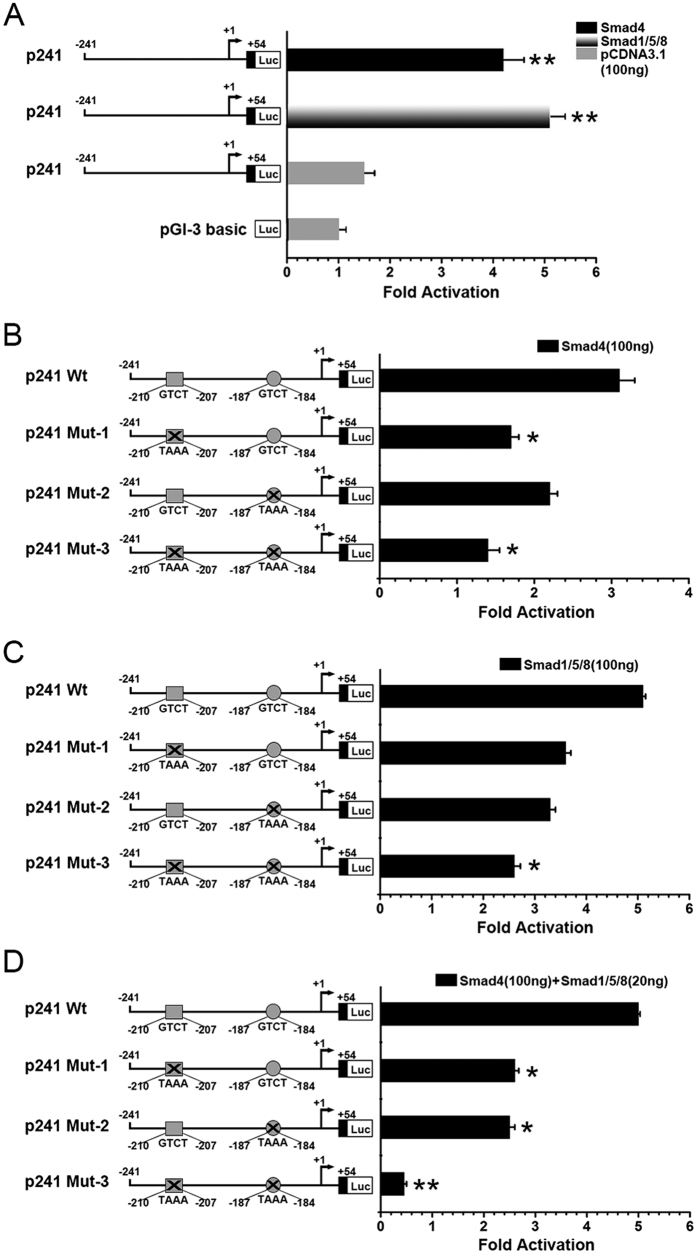
DSP ^aa183-219^ up-regulates DSPP gene transcription through SMAD1/5/8. (**A**) pGL-Luc-mDSPP-241/+54 (p241) were co-transfected with 100 ng of pcDNA-Smad4, pCMV-Smad1/5/8, or pcDNA3.1. The transcription results were computed as luciferase activities per mg of total protein. The value obtained from the control group was considered as 1-fold. Fold increases were calculated by dividing the individual value by the control group. (**B**) p241 Wt, p241 Mut-1, p241 Mut-2, or p241 Mut-3 were co-transfected with 100 ng pcDNA-Smad4. (**C**) p241 Wt, p241 Mut-1, p241 Mut-2, or p241 Mut-3 were co-transfected with 100 ng pcDNA-Smad1/5/8. (**D**) p241 Wt, p241 Mut-1, p241 Mut-2, or p241 Mut-3 were co-transfected with 100 ng pcDNA-Smad4 and 20 ng Smad1/5/8. The data are the mean ± S.D. from independent experiments performed in triplicate.

To determine the biological function of -211 to -183 of the mouse DSPP promoter region, the DSPP promoter -241/+54 (p241) was subcloned into a Luc-report vector (pGL-Luc-mDspp-241/+54). For determination of transcriptional activity, the pGL-Luc-mDSPP-241/+54 vector was transfected into 293T cells, and its transcriptional activity was determined in the presence of pcDNA-SMAD4, pCMV-SMAD1, pCMV-SMAD5, pcDNA-SMAD8, or pcDNA3.1 after 48 h of co-transfection. Both SMAD4 and SMAD1/5/8 stimulated an increase in mDSPP-241/+54 promoter activity (Fig. 7A). Promoter activity stimulated by SMAD1/5/8 was high compared to the group with SMAD4 induction. Two SBE sites are located in this -241/+54 region. To study the biological functions of these two sites, we generated three mutant DNA constructs: p241 Mut-1, p241 Mut-2, and p241 Mut-3 (Fig. 7B). The promoter activities of the p241 mutant constructs were approximately 50% reduced compared with those of p241 Wt when 100 ng of either SMAD4 or SMAD1/5/8 was present, and both of the SBEs had a synergic effect on DSPP gene transcription (Fig. 7B,C). To further study the synergic effect of SMAD4 and SMAD1/5/8 on DSPP gene expression, SMAD4 and SMAD1/5/8 expression vectors were co-transfected with p241 Wt or p241 mutant constructs. Our results showed that SMAD4 combined with SMAD1/5/8 dramatically increased DSPP reporter activity compared with that of only SMAD4 or SMAD1/5/8 (Fig. 7B–D).

## Discussion

Previous studies have suggested that DSP plays a role in regulating the initiation of dentin mineralization. DSP increases the rate of enamel mineralization and induces dental pulp cell differentiation and mineralization^13^,^30^. Mutations of the DSP domain cause DGI-II, DGI-III and DD-II^7^,^8^,^9^,^10^,^11^. We and other laboratories reported that full-length and COOH-terminal DSPs regulate bone/tooth related gene expression and stimulate kinase phosphorylation and dental mesenchymal differentiation^29^,^30^. However, the molecular mechanisms of DSP in regulating intracellular signal pathways remain unclear. Here, for the first time, we found that the DSP ^aa183-219^ fragment acts as a ligand and binds to cell surface receptor integrin β6 in an RGD-independent manner and regulates cell behaviors via the P38-ERK-SMAD1/5/8-DSPP signal pathway.

Although DSPP is transcribed as a single gene, full-length DSPP protein rarely exists in odontoblasts and dentin, but is often processed into DSP, DGP and DPP^3^,^18^. Both DSP and DPP play distinct roles in dentinogenesis^6^. DSP is further processed into small molecular fragments, and these processed DSP fragments are distributed into different compartments of the tooth^38^. SIBLING family members contain a tripeptide RGD and RGD motif within the SIBLING genes that binds to integrins, activating intracellular signal transduction^22^,^23^,^25^,^26^,^27^,^39^. However, DSP lacks the RGD motif. Therefore, how DSP signal affects intracellular signal pathways has not well been characterized. In the present study, we uncovered that, as a ligand, DSP was capable of interacting with integrin β6. Furthermore, we found that the 36-amino acid residues of DSP ^aa183-219^ were sufficient to bind integrin β6. Similar to full-length DSP, DSP ^aa183-219^ was sufficient to induce dental papilla mesenchymal cell proliferation and differentiation. In addition, this peptide promoted cell attachment, spreading and migration. More importantly, the DSP ^aa183-219^ fragment up-regulated endogenous DSPP and DMP1 gene expression in mouse dental papilla mesenchymal cells. These results indicate that the DSP ^aa183-219^ fragment induces differentiation of dental papilla mesenchymal cells into odontoblast-like cells via a positive forward signaling loop. The molecular mechanisms of DSP ^aa 183-219^ involve the mediation of P38, ERK1/2 and SMAD1/5/8 phosphorylation signaling in mDPC6T cells. Cell attachment, spreading, migration and differentiation mediated by DSP ^aa183-219^ are inhibited by the inhibitors against integrin β6, SMAD1/5/8, P38 or ERK1/2.

Integrin β6 interacting with ECM ligands activates a series of protein kinases, such as ERK1/2, via matrix-cell interactions (50–53). Activated protein kinases further stimulate the activity of transcription factors, which are translocated into nuclei and bind to their binding site (s) in target gene regulatory regions, thus activating the transcription of these gene (48, 49). In this study, we found that DSP ^aa183-219^ stimulated phosphorylation of P38 and ERK1/2 through integrin β6 and that the effect of DSP ^aa183-219^ on the phosphorylation of these kinases was inhibited by an anti-integrin β6 antibody and siRNA. Additionally, we observed that P38 and ERK1/2 phosphorylation induced by the DSP ^aa183-219^ fragment occurred earlier than that of SMAD1/5/8 in dental papilla mesenchymal cells and that inhibition of P38 and ERK1/2 phosphorylation resulted in decreased phosphorylation and nuclear translocation of SMAD1/5/8. Therefore, the DSP ^aa183-219^-integrin β6 complex promotes phosphorylation and nuclear translocation of SMAD1/5/8 mediated by the P38-ERK1/2 signaling pathway.

The SMAD pathway is the canonical signaling pathway activated directly by TGF-β/BMP cytokines. These cytokines are divided into R-SMADs, Co-SMADs, and I-SMADs. R-SMADs are further divided into SMAD2/3 and SMAD1/5/8. SMAD2/3 is activated by TGF-β, whereas SMAD1/5/8 is activated by BMPs^40^. Previous studies have reported that expression of SMADs is detected in tooth germ epithelium and mesenchyme^41^. In this study, expression of SMAD1/5/8 is detected in mDPC6T cells. Studies have indicated that SMAD1/5/8 phosphorylation is activated by BMP2 or full-length DSP in the context of odontoblast differentiation^30^,^42^. C2C12 cell differentiation into osteoblasts induced by BMP2 is inhibited when SMAD1/5/8 is blocked^43^,^44^. The mediating factor SMAD4 also plays a role in dentinogenesis. Inhibition of SMAD4 leads to odontoblast defect and irregular dentin^45^. In addition, MAPK phosphorylates SMAD1/5/8^32^,^46^. In mDPC6T cells, DSP ^aa183-219^ binds to integrin β6 and causes activation of ERK1/2 and P38, which initially phosphorylates SMAD1/5/8 and ultimately induces odontoblast differentiation mediated by increased DSPP and DMP1 expression. We found that phospho-SMAD1/5/8 is inhibited by P38 and ERK inhibitors (SB203580, PD98059). Therefore, we revealed that SMAD1/5/8 in coordination with SMAD4 promotes DSPP gene expression and odontoblast differentiation through a non-canonical signaling pathway.

To investigate whether DSPP gene expression is controlled by SMAD1/5/8, we identified SBEs in the DSPP gene promoter using a computer-aid software program and performed DNA-protein binding assays. EMSA and ChIP assays revealed that SMAD4 and SMAD1/5/8 bind to SBEs in the DSPP regulatory region. Super shift and competition assays further confirmed these results. A biological function study demonstrated that SMAD1/5/8 and SMAD4 enhance DSPP gene promoter activity via SBEs. Mutations of SBEs in the DSPP gene promoter dramatically decrease DSPP gene transcription. In addition, both of SMAD1/5/8 and SMAD4 have synergic effects on DSPP gene expression.

**Figure 8:**
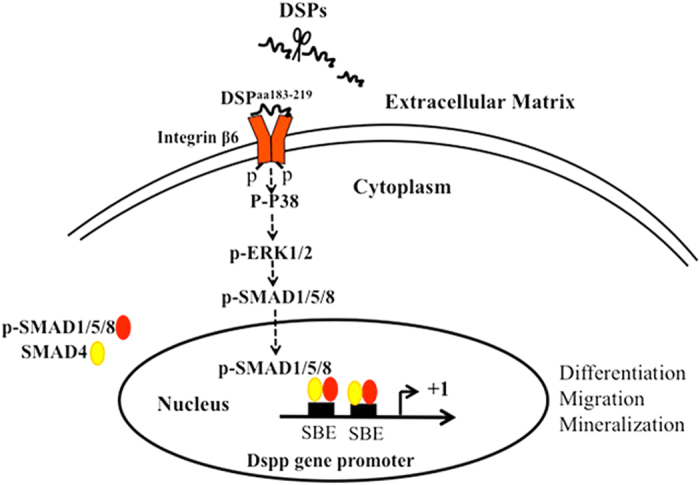
Regulation of cell differentiation and mineralization by the DSP-integrin β6 protein complex. DSP ^aa183-219^ interacts with integrin β6 and forms a complex, activating phosphorylation of P38, ERK1/2 and SMAD1/5/8. Phosphorylated SMAD1/5/8 proteins are translocated into nuclei. SMAD1/5/8 in coordination with SMAD4 bind to SMAD binding elements (SBEs) in the DSPP gene promoter and stimulate DSPP gene transcription. Therefore, the DSP-integrin β6 signal positively promotes up-regulation of DSPP expression and dental mesenchymal cell attachment, spreading, proliferation, differentiation and migration.

In conclusion, we found that the 36-amino acid residues of DSP ^aa183-219^ act as a ligand and bind to its receptor integrin β6. The DSP-integrin β6 complex activates phosphorylation of P38, ERK1/2 and SMAD1/5/8. Phosphorylated SMAD1/5/8 trans-localizes into nuclei and binds to SBEs in the DSPP gene promoter, activating DSPP gene transcription. Therefore, DSP ^aa183-219^ promotes dental mesenchymal cell proliferation, migration, differentiation and mineralization through integrin β6-P38-ERK1/2-SMAD1/5/8-DSPP signaling. DSP ^aa183-219^ regulates odontoblast homeostasis via a positively forward pathway loop. Figure 8 depicts a model by which DSP regulates dental cell differentiation and mineralization via integrin β6/P38/ERK1/2/SMAD1/5/8/DSPP signaling based on this study.

## Materials and Methods

### Antibodies and Reagents

Anti-phospho-ERK1 (T202/204)/ERK2 (T185/Y187) and anti-phospho-P38 MAP Kinase (T180/Y182) antibodies were purchased from R&D (USA). The anti-phospho-SMAD1/5/8 (pS463/465) antibody was purchased from Epitomics (USA). Anti-ERK1/2, anti-P38 and anti-SMAD1/5/8 antibodies were purchased from Cell Signaling Technology (USA). Anti-integrin β6, anti-DSP (M300 for N-terminal of DSP), anti-DSP (M20 for C-terminal of DSP), anti-SMAD4 and anti-β-actin antibodies were purchased from Santa Cruz Biotechnology, Inc. (USA). The anti-GST antibody was purchased from GE Healthcare (USA). The anti-Myc antibody was purchased from Thermo Scientific (USA). The anti-FLAG antibody was purchased from Sigma-Aldrich (USA). The anti-DMP1 antibody was purchased from EMD Millipore (USA). SB203580 (P38 inhibitor) was purchased from Cell Signaling Technology (USA). PD98059 (MAP Kinase Inhibitor) was purchased from Invitrogen (USA). The integrin β6 and SMAD1/5/8 silencer select pre-designed siRNAs were purchased from Ambion (USA).

### Plasmids

The constructs were prepared according to standard techniques. The DSP expression plasmids were constructed by cloning different domains of the DSP open reading frame from plasmid pBluescript-SK-DSPP using PCR and then inserting the resultant DNA sequence into the pGEX-6P vector. For the GST pull-down assay or co-immunoprecipitation (co-IP) assays, DSP fragments encoding amino acid residues 9–456, 9–190, 183–456, 183–295, 266–371, 365–456, 183–219, 214–246, 240–271, or 266–299 were amplified by PCR using appropriate sets of primers, and then PCR products were inserted into pGEX-6P or pFLAG-CMV-2. All constructs were verified by DNA sequencing. The plasmids encoding full-length integrin β6, SMAD4, SMAD1, SMAD5, and SMAD8 were purchased from Addgene (USA).

### GST fusion proteins and pull-down assay

For bacterial expression of recombinant DSP (rDSP), the pGEX-6p-DSP plasmids were transformed into *Escherichia coli* BL21. Expression of the GST fusion protein was induced with 1 mM IPTG at 37 °C for 5 h. The rDSP was purified with glutathione-agarose beads (Sigma-Aldrich, USA). The purified protein concentration was detected with a Micro BCA^TM^ Protein Assay Kit according to the manual instruction. The purified rDSP was confirmed and verified with Coomassie blue staining and western blot assay using anti-DSP and anti-GST antibodies.

### Cell culture and transfection

mDPC6T cells are an immortalized mouse dental papilla mesenchymal cell line as described previously^47^. The cells were grown in DMEM containing 10% fetal bovine serum plus penicillin (100 U/ml) and streptomycin (100 mg/ml) and were cultured at 37 °C in a humidified atmosphere of air containing 5% CO_2_. The medium was refreshed every 2 days. Human embryonic kidney 293T cells (Invitrogen, USA) were maintained in DMEM supplemented with 10% FBS plus penicillin (100 U/ml) and streptomycin (100 mg/ml) and cultured at 37 °C in a humidified atmosphere of air containing 5% CO_2_. For western blotting and immunocytochemical assays, mDPC6T cells were cultured in medium without serum for 12 h prior to treatment with DSP ^aa183-219^ (200 ng/ml). Cells were cultured for 0, 2, 5, 15, 30, 60, and 120 min prior to protein analysis. Then, 293T cells were seeded in 100-mm dishes and allowed to proliferate until 70% to 90% subconfluent before transfection. Cells were then cultured for an additional 48 h prior to protein extraction.

### Co-immunoprecipitation assay

The FLAG-DSP and Myc-integrin β6 plasmids were transfected into mDPC6T cells from a 100-mm Petri dish (70–90% subconfluent) using Thermo Scientific TurboFect Transfection Reagent (Thermo Scientific, USA). The cells were lysed in 1 mL of cold lysis buffer containing 50 mM Tris-HCl, pH 7.4, 150 mm NaCl, 1 mM EDTA, 1% TRITON X-100 (Sigma-Aldrich, USA) and protease inhibitor cocktail (Sigma-Aldrich, USA) and mixed with a vortex mixer for 1 h at 4 °C. Insoluble materials were removed by centrifugation at 12, 000 g for 10 min at 4 °C. Lysates containing 4 mg of protein in 1 mL were precleared by incubating with 40 ul anti-FLAG or anti-Myc Affinity Gel (Sigma-Aldrich, USA) at 4 °C on a rotator overnight. Immunoprecipitates were washed three times with TBS containing 50 mm Tris⁄150 mm NaCl, pH 7.4, and heated for 5 min at 95 °C in 20 μl 2× SDS-PAGE sample buffer (62.5 mM Tris HCL, pH 6.8. with 2% SDS, 10% glycerol, 0.002% bromophenol blue) and prepared for western blotting.

### Cell attachment and spreading analysis

Briefly, 96-well tissue culture plates (Corning, USA), were coated with 200 ng/ml of DSP ^aa183-219^ in 20 mM carbonate buffer, pH 9.3 at 4 °C for 2 days. Then, the DSP ^aa183-219^-coated plates were blocked with 1% (w/v) bovine serum albumin (BSA) for 1 h at room temperature. mDPC6T cells (2 × 10^3^ cells/per well) were seeded to the plates and incubated for 1.5 h. Non-adherent cells were gently washed from the wells with PBS, and the adherent cells were allowed to grow overnight. The attached cells were fixed and stained with 0.1% (w/v) crystal violet (Sigma-Aldrich, USA) for 15 min at 37 °C. After washes with distilled water, attached cells were counted under a microscope. The cells grown on BSA-coated plates served as a control. For cell spreading assays, mDPC6T cells were seeded on DSP ^aa183-219^- or BSA-coated cover glass for 1, 2, 4, 8, and 24 h. The cells were fixed and stained for actin.

### Cell migration assay

Cell migration assays were performed using cell culture inserts incorporating polyethylene terephthalate (PET) track-etched membranes with 8-μm perforations (BD Biosciences, USA). The cell culture inserts were placed in 12-well plates. Cells (10^5^ cells/well) were added into the upper chamber in 300 μl of DMEM containing 5% FBS. The lower chamber contained DMEM with 200 ng/ml or 1 μg/ml of DSP ^aa183-219^ in 5% FBS as a chemoattractant. After a 12-h incubation of 5% CO_2_ at 37 °C, the filters were fixed and stained with 0.1% (w/v) crystal violet (Sigma-Aldrich, USA) for 15 min at 37 °C. The number of cells that migrated through the filters was quantified by counting 10 fields per membrane at a 200-fold magnification. The lower chamber contained DMEM with or without BSA in 5% FBS as a control. Cells were seeded at a density of 5 × 10^4^ cells/well and grown to confluence. Cell monolayers were “scratched” with a sterile pipette tip to create an area devoid of cells, which was rinsed twice with PBS. The location of the “scratch” was marked on the underside of the dish using a marker pen. Cells were incubated with or without 10 μg/ml DSP ^aa183-219^. Cell migration was visualized under a microscope at 0, 12, 24, and 36 h after the scratch was generated^48^.

### Cell proliferation assay

Cell proliferation assays were performed by direct cell counting. Briefly, cells were seeded into 6-well plates at 10^4^ cells per well and then treated with or without 200 ng/ml of DSP ^aa183-219^ at days 1, 3, 6, 9, 12, 15 and 18. The cells were trypsinized and counted using a hemocytometer.

Alkaline phosphatase (ALP) activity assay—mDPC6T cells were treated with or without 200 ng/ml of DSP ^aa183-219^ in 6-well plates at a density of 4 × 10^5^ per well and cultured in calcifying medium (α-MEM supplemented with 10% FBS, penicillin (100 U/ml) and streptomycin (100 μg/ml), 50 μg/ml ascorbic acid, 0.1 μM dexamethasone and 10 mM sodium β-glycerophosphate) at 37 °C for 1, 3, and 7 days. The cell lysates was assayed using *p*-nitrophenylphosphate as a substrate. The protein concentration was determined using the bicinchoninic (BCA) protein assay reagent (Thermo Scientific, USA). The enzyme activity was expressed as nanomoles of *p*-nitrophenol produced per gram of protein in 15 min.

### Alizarin red S staining

mDPC6T cells were treated with or without 200 ng/ml of DSP ^aa183-219^ in 6-well plates at a density of 4 × 10^5^ per well and cultured in calcifying medium at 37 °C for 7, 14, and 21 days. Cells were fixed in 95% methanol for 45 min at 4 °C followed by washing with H_2_O. The cultures were then stained with 0.5% Alizarin red S (Sigma-Aldrich, USA) for 30 min at 37 °C, washed with H_2_O, and observed under a microscope.

### Quantitative real-time PCR (qRT-PCR) analysis

Cells were cultured in complete basal medium in 6-well plates to 70% to 80% confluence. Cells were then treated in triplicate with DSP ^aa183-219^ for 0, 6, 12, 24, and 48 h. Total RNA was extracted using TRIZOL reagent. After RNA was extracted, qRT-PCR was performed as described previously^49^. The following PCR conditions were employed: step one, 94 °C for 3 min, 1 cycle; step two, 94 °C for 30 s, 60 °C for 30 s, 72 °C for 30 s, 34 cycles; step three, 72 °C for 10 min; step four, 4 °C forever. Target gene primers used for qRT-PCR are presented in Table 1. Gene expression levels were calculated as fold changes compared with basal medium control^50^.

### Immunocytochemistry

Cells were cultured on glass coverslips in 6-well plates. Cells were then treated with 200 ng/ml of DSP ^aa183-219^ for 0, 2, 5, 15, 30, 60, and 120 min. All subsequent steps were performed at room temperature. Cells were then rinsed twice with PBS and fixed in 4% paraformaldehyde for 10 min. Fixed cells were rinsed twice in PBS and then permeabilized with 0.1% Triton X-100 in PBS for 5 min. After washing, cells were incubated in 2.5% BSA/PBS for 1 h and followed by a primary antibody (p-ERK, p-P38, p-SMAD) at 4 °C overnight. Cells were washed three times in wash buffer and incubated with a Dylight 488-conjugated secondary antibody (goat anti-rabbit) for 1 h. Cells were washed three times with wash buffer. Coverslips were inverted and mounted on glass microscope slides using DAPI mounting medium and visualized using a fluorescence microscope.

### Western blot analysis

Total proteins were extracted from mDPC6T cells treated with or without DSP ^aa183-219^. A total of 30 μg of protein was resolved by 10% SDS-PAGE. After electrophoresis, the proteins were transferred onto a nitrocellulose membrane (Bio-Rad, USA), blocked with 5% BSA in PBS, and probed with anti-DSP (1:1000), anti-β6 (1:1000), anti-ERK1/2 (1:1000), anti-phospho-ERK1/2 (1:1000), anti-P38 (1:1000), anti-phospho-P38 (1:1000), anti-DMP1 (1:4000), anti-FLAG, anti-Myc, or anti-GST antibodies. HRP-conjugated goat anti-rabbit IgG was used for detection (Chemicon International Inc., USA).

### Electrophoretic mobility shift assay (EMSA)

EMSA was performed according to Chen *et al*.^51^. Briefly, nuclear extracts (5–10 μg) were preincubated in a total volume of 20 μl of binding buffer (10 mM Tris-HCl, pH 7.5, 50 mM NaCl, 1 mM EDTA, 1 mM dithiothreitol, 5% glycerol) containing 2 μg of poly (dIdC) for 5 min at room temperature. After pre-incubation, ^32^P-end-labeled DNA fragments (1 ng) were added and incubated for an additional 20 min. For competition-binding reactions, unlabeled DNA fragments in 100-fold or 300-fold molar excesses of the labeled DNA probe were added into the reaction. The products of the DNA-protein reaction were separated by electrophoresis on a non-denaturing 5% polyacrylamide gel in 1× TBE buffer. DNA-protein complexes and unbound DNA probe were visualized on X-ray film. For gel mobility supershift analysis, anti-SMAD4 or anti-PSMAD1/5/8 antibodies (Santa Cruz Biotechnology Inc., USA) were preincubated with nuclear extracts 10 min prior to the addition of the radiolabeled probe. The following reactions were performed as described above.

### Chromatin immunoprecipitation assays

Briefly, 293T cells grown in a 10-cm dish were transiently transfected with pcDNA-Smad4, pCMV-Smad1, pCMV-Smad5, pcDNA-Smad8, and pGL-Luc-mDSPP-500/+54 using Lipofectamine 2000 (Invitrogen, USA). Chromatin immunoprecipitation (ChIP) assays were performed according to the manufacturer’s instructions using the ChIP Assay Kit (EMD Millipore, USA). Briefly, 48 h after transfection, the cells were crossed-linked with 1% formaldehyde for 10 min, washed with cold PBS and lysed in SDS lysis buffer. Lysates were sonicated to shear DNA, and the supernatant was diluted 10-fold in ChIP dilution buffer. A proportion of the diluted supernatant was kept as input DNA. After preclearing for 30 min with protein A agarose/salmon sperm DNA, samples were incubated with an anti-pSMAD1, anti-pSMAD5, anti-pSMAD1/5/8, or anti-SMAD4 antibody overnight at 4 °C. Negative control IgG was included. Protein A agarose/salmon was then added for 1 h at 4 °C to collect the immune complexes. Then, the immune complexes were sequentially washed with low salt, high salt, LiCi immune complex washing buffers and TE buffer. Immune complexes were eluted by the addition of elution buffer for 30 min with rotation at room temperature. Cross-links were reversed by the addition of 5 M NaCl and heating at 65 °C for 4 h. Samples were then incubated with 0.5 M EDTA, 1 M Tris-HCl and 10 mg/ml proteinase K for 1 h at 45 °C. DNA was then recovered by phenol/chloroform extraction, precipitated with ethanol and resuspended in 20 μl of water. Recovered DNA was analyzed by PCR using the following primers: forward, 5′-AAATGCAGGGTGACAGAGTCTAAGT-3′; reverse, 5′-ATAGGCACACTGACTCTTTAAACCC-3′. This pair of primers was designed to amplify the mouse DSPP gene promoter region from -326 to -113.

### Luciferase reporter assay

Cells grown in 24-well plates were transfected with pGL3-Basic empty vector or pGL3-Luc-mDSPP-241/+54 and pRL-TK *Renilla* luciferase expression vector as an internal control as well as pcDNA-Smad4, pCMV-Smad1, pCMV-Smad5, pcDNA-Smad8, or pcDNA3.1 using Lipofectamine 2000. Cells were collected 48 h after transfection and lysed in passive lysis buffer. The luciferase assay was performed using a Dual Luciferase Reporter Assay System (Promega, USA) according to the manufacturer’s instructions. Firefly and *Renilla* luciferase activities were determined using the Glomax Luminometer (Promega, USA). Luciferase expression was normalized against *Renilla* luciferase expression to determine relative luciferase activity.

### Statistical analysis

Quantitative data are presented as the mean ± S.D. from three independent experiments. Statistical analysis was performed with a two-tailed *t* test using SPSS statistics 17.0. *P*-values < 0.05 were considered statistically significant.

## Additional Information

**How to cite this article**: Wan, C. *et al*. The Dentin Sialoprotein (DSP) Domain Regulates Dental Mesenchymal Cell Differentiation through a Novel Surface Receptor. *Sci. Rep.* **6**, 29666; doi: 10.1038/srep29666 (2016).

## Figures and Tables

**Table 1:** Oligonucleotide Primer Sequences Used in the qRT-PCR.

Gene	Sequence (5′-3′)	Size (bp)
Alkaline phosphatase (ALP)	Forward: CTGATGTGGAGTATGA	96
	Reverse: TGTATCTCGGTTTGAA	
Dentin matrix protein-1 (DMP-1)	Forward: ACAGGCAAATGAAGACCC	152
	Reverse: TTCACTGGCTTGTATGG	
Dentin sialophosphoprotein (DSPP)	Forward: TGCTGGAGCCACAAAC	124
	Reverse: AAACCCTATGCAACCTTC	
Glyceraldehyde-3-phosphate (GAPDH)	Forward: TGCACCACCAACTGCTTAGC	78
	Reverse: GGCATGGACTGTGGTCATGAG	
